# Supplementary material for: Natural 2-Amino-3-Methylhexanoic Acid as Plant Elicitor Inducing Resistance against Temperature Stress and Pathogen Attack
Source: Int J Mol Sci. 2022 May 20;23(10):5715. doi: 10.3390/ijms23105715 (PMC9147978; doi:10.3390/ijms23105715)
Supplement: Supplementary file 1 [file ijms-23-05715-s001.zip › ijms-1709660-supplementary.pdf]

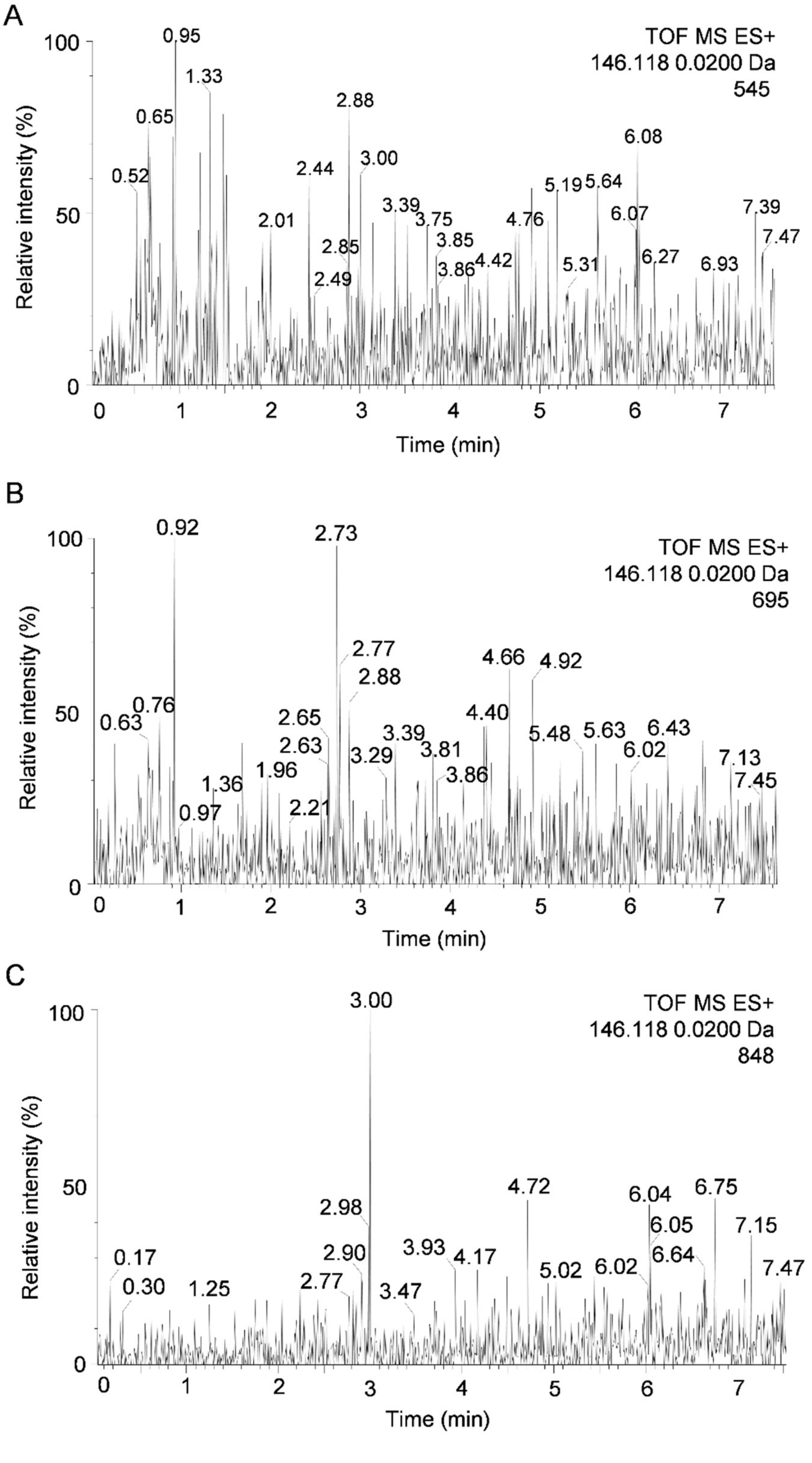

Figure S1

Test of AMHA in leaves of three different plants. (A) UPLC-MS chromatograms in positive ion mode of the extracts from 21-day-old leaves of *Arabidopsis* (A), leaves of two-leaf stage seedlings of winter wheat (B) and rice (C). Results represent three independent biological replicates.

Table S1.

Effect of AMHA on the growth of different fungal pathogens on PDA medium.

Fungus species	Diameter of colony (mm)			
	Mock (0)	0.5 mM AMHA	1 mM AMHA	2 mM AMHA
Magnaporthe oryzae	64.15±0.89 a	62.84±0.38 a	63.36±0.48 a	61.04±0.11 a
Rhizoctonia solani	68.06±0.36 a	68.01±0.45 a	67.57±0.48 a	67.83±0.03 a
Fusarium moniliforme	65.41±0.22 a	63.65±0.51 a	63.30±0.39 a	61.98±0.23 a
Fusarium graminearum	73.12±0.37 a	69.35±0.16 a	66.69±0.40 a	63.62±0.30 b
Rhizoctonia cerealis	56.46±0.83 a	56.23±0.40 a	55.62±0.27 a	51.08±0.81 a
Botrytis cinerea	68.71±0.13 a	69.06±0.39 a	68.65±0.31 a	68.07±0.34 a
Sclerotinia sclerotiorum	62.61±0.38 a	63.59±0.40 a	64.45±0.34 a	62.58±0.87 a
